# Implementing ideal health policy in a fragile health system: the example of expanding the use of malaria rapid diagnostic tests in mainland Tanzania

**DOI:** 10.1186/1475-2875-10-322

**Published:** 2011-10-28

**Authors:** Irene M Masanja, Xavier de Bethune, Jan Jacobs

**Affiliations:** 1 Health Systems Group, Ifakara Health Institute, Kiko Avenue- Mikocheni, PO Box 78373, Dar es Salaam, Tanzania; 2 Emerging Voices programme (2010), Institute of Tropical Medicine, Nationalestraat 155, 2000 Antwerp, Belgium; 3 Christian Sickness Fund, Chaussée de Haecht 579, BP 40, B-1031 Brussels, Belgium; 4 Clinical Sciences Department, Institute of Tropical Medicine, Nationalestraat 155, 2000 Antwerp, Belgium

**Keywords:** Malaria rapid diagnostic tests, health systems, Tanzania

## Abstract

Malaria confirmation before treatment provides an opportunity for improving the quality of malaria case management in endemic regions. However, increased coverage of this strategy is facing many organizational, logistical and technical challenges that threaten its success. Introducing an intervention with system-wide effect, such as the use of malaria rapid diagnostic tests in areas where malaria is still a public health problem, should be accompanied by system strengthening measures to better attain the goal of improving quality of care.

## Background

In recent papers in Malaria Journal, Graz *et al *[1] and D'Acremont *et al *[2] provided a good insight on the policy of malaria confirmation prior to treatment in areas with different transmission intensities. Graz *et al *[1] questioned WHO recommendations of the 'test and treat' strategy, whereas D'Acremont *et al *demonstrated how programmatic implementation of malaria rapid diagnostic tests (RDTs) in moderately malaria endemic areas drastically reduced over-treatment with anti-malarials [2]. These articles give a clear viewpoint of the future of malaria diagnosis in endemic countries, particularly in sub-Saharan Africa. Given the growing evidence of advantages acquired by introduction of malaria RDTs, it is to be expected that most malaria endemic countries will opt for a confirmatory policy. Apart from the issue of different policies in malaria diagnosis cited by Graz and coworkers, other issues are to be considered in order to ensure successful adoption of such policy. In particular, the countries' health systems must have appropriate resources and skills in place.

Increased coverage of malaria confirmation prior to treatment by use of RDTs represents renewed hope for quality management of malaria cases. In Tanzania, a country with high to low malaria transmission, RDTs are currently being rolled out. The advantages of confirming malaria before treatment has been extensively explored by both Graz and D'Acremont and colleagues [1,2]. The fact that RDTs will be made available to the dispensaries-the lowest level of the formal health care system in Tanzania, closer to the population-ensures that the strategy will be widely accessible. However, the strategy is faced with many organizational, logistical and technical challenges. The introduction of RDTs in Tanzania has uncovered many health system bottlenecks that necessitate the development of new approaches to deliver quality health care. The World Health Organization (WHO) health systems building blocks [3] provide a good framework to assess these effects. This commentary is trying to highlight some aspects of these challenges and possible ways of minimizing them, through an informal observation of the implementation in mainland Tanzania of an RDT policy in place since 2008. In this policy, RDTs are to be used for malaria confirmation at all levels of care [4] throughout the year, complementing microscopy where present (National Malaria Control Programme, Dar es Salaam, Tanzania: National Guidelines for the use of Rapid Malaria Diagnostic Tests in Tanzania. 2007). The need for confirmatory tests in the country is justified now because malaria prevalence is on the decline [5].

### Service delivery-treatment based on test results

Early experiences of implementing the malaria confirmation policy in Tanzania revealed some undesirable prescription behaviours, *i.e*. clinicians continued to prescribe anti-malarials despite a negative test result. This problem has previously been reported in Tanzania [6] and poses a serious concern about appropriate malaria case management in such a resource-poor setting. Bell and Perkins [7] highlighted the consequences of implementing RDTs as a tool for malaria control rather than fever management. In their review, they pointed out a long-held belief that fever without obvious cause equals malaria, and this attitude may still make both providers and clients reluctant to trust negative RDT results.

The success of RDT implementation with resultant marked reduction of anti-malarial usage, as demonstrated by D'Acremont and colleagues and Masanja *et al*, is encouraging [2,8], although the need for monitoring antibiotic use has become more apparent. Nevertheless, behaviour change is a lengthy process. When planning an increased coverage of RDTs, enough emphasis should thus be placed on prescription practices. This can, for instance, be done through discussion platforms for clinicians about alternative management of fever with a negative malaria test. The knowledge of local epidemiology as stated by Graz *et al *is important [1], but requires knowledge of local disease profiles and capacity to confirm-"rule-in" or "rule-out".

### Health workforce

In rural areas a health worker who performs RDTs often also performs patients' consultations, dispenses drug or deals with routine maternal and child health services, including vaccinations, family planning or delivery. It is not uncommon to find only a single provider combining these health care services in a remote facility. Malaria being a leading cause of hospital attendance [9], any added procedure in the line of clinical care will without doubt increase the time a clinician spends to manage patients and thus add to the health workers' workload. The critical shortage of health workforce in Tanzania demands strong decisions to improve health care services.

For example, compelling evidence shows that health extension workers, such as community or village health workers, can perform non-specialized clinical work including RDT testing with high accuracy [10,11]. To deal with the problem of workload following the introduction of RDTs, the Tanzanian government could either consider bringing back village health workers-after having addressed the issues that led to the failure of this programme in the 1980s-or increase output from medical and paramedical schools as well as create more employment opportunities in the health sector. This may sound as a far-fetched dream for the time being, but its relevance will be apparent once health providers are fatigued with the new intervention and stop testing patients. In a pragmatic implementation, as was the case for D'Acremont *et al *[2] and Masanja *et al *[8], the study team works closely with health workers, therefore, a relatively better compliance should be expected.

The move made by the Tanzanian Ministry of Health to adapt RDTs standard operating procedures (SOP) to the local environment, based on the generic SOP provided by WHO, proved to be very useful in training lower health cadres to perform the test. Clarity of written instructions has been shown to improve health workers performance of malaria rapid tests in other malaria endemic areas [12].

### Medical products, vaccines and technologies-availability of testing devices

Even in the early phases of the Tanzanian nation-wide roll out of RDTs, unexpected stock-outs of test kits occurred in health facilities. Delays in donor funds' disbursement, caused by delays in publication of national audit and consumption reports, contributed to these stock-outs. The problems with the consumption reports are primarily a result of insufficient facility-based utilization data. One way of minimizing this problem is to expand the use of consumption data from health facilities as is the case with 'SMS for Life' programme. In the SMS for Life, various stakeholders teamed up to provide timely access to anti-malarial drugs in rural areas by monitoring drug stocks in health facilities using mobile phones. This strategy is now being adopted and scaled-up by the Tanzanian government to monitor anti-malarial stocks.

Dispensaries being the closest structures to the community are not designed to provide laboratory services. Moreover, RDTs require specific procurement, stock management and storage conditions. Accordingly, the introduction of RDTs at dispensary level should be a very thoroughly planned programme of diagnostic services expansion. Having different policies for a centrally controlled intervention will complicate its functioning and the supervision of national guidelines, which the governing boards strives to standardize.

### Information flow and governance

In Tanzania, procurement of RDTs is done centrally by the Medical Stores Department, upon receiving advance payment from the Ministry of Health and Social Welfare (MoHSW). Any delays in funds disbursement *e.g*. from the Treasury to the MoHSW slows down the whole process of ordering and procuring tests devices. As a result, district authorities are left to manage unequipped health facilities. This communication breakdown complicates the existing management systems between central and local governments. The local government authority through the District Health Management Team is responsible for the performance of district health workers while the central government formulates policy and monitors its implementation. Only good governance ensures the smooth running of activities.

Masanja *et al *[8] reported good clinicians' acceptance of RDT test results and adherence to national treatment guidelines in their study setting, mostly fostered by adequate supervision and on the job training. This shows that appropriate supervision and good governance can bring about major improvements in system performance. An adequate flow of information from central to local government (*e.g*. about major delays of RDT shipment) and vice versa (*e.g*. timely provision of health facility data for national audit reports) will reduce delays both ways. Where support supervisions happen, appropriate feedback aiming to improve performance can be provided. Learning is best done by participation. A well functioning health information system should allow constant follow up of system performance, guide resource allocation and help decision-making processes.

### Financing (and people centeredness)

Health system performance is difficult to predict and people should be regarded as core actors. Negative perceptions of people will undoubtedly affect the success of any policy implementation. For example, in Tanzania there are variations of malaria transmission intensity with areas of high, moderate and very low transmission. In this respect, population appreciation of the malaria problem may differ and affect the acceptability of malaria test results. This can be a problem to providers and the general public, hence requires a good RDT quality assurance system to monitor performance and accuracy. Any mistrust of the new tests among the public will affect their acceptability, thus failing to protect people from financial expenditure due to searching of quality care including parasitological confirmation elsewhere.

Catastrophic expenditure related to malaria care-seeking has long been reported for the poorest quintile of the population. The social and economic burden of malaria at individual, household and national levels has been extensively studied [13-15]. In the Tanzanian example, it is unfortunate that substantial public sensitization about the introduction of RDTs has yet to happen, thus the public is less informed about the government's decision to introduce RDTs in the line of care and their expected benefits.

### Systems strengthening

It is imperative to understand that health systems are very unique and highly context specific. Most health care systems in developed countries which are able to provide quality care share some fundamental characteristics, such as good interaction amongst key actors, integration of service delivery packages, trained manpower, appropriate supervision, timely availability and accessibility of health services. This remains a major challenge in most malaria endemic countries. The fact that most malaria stricken countries in sub-Saharan Africa still have lackluster economic growth implies that having an appropriate health policy in place not necessarily guarantees improved quality of care. There are other major obstacles to be overcome for the new policy to bring about the desired effects. Realization of the need to empower the health system to adapt the new intervention in the Tanzanian example came after the policy change. However, early experiences provided the best opportunities to assess implementation gaps and start addressing them.

## Conclusion

A perfect health policy in place will not always guarantee improved care. System strengthening should form a major component of proposed interventions expected to bring about system-wide effects in low-income countries with weak and fragile health systems. A policy of malaria confirmation before treatment in endemic countries, with increased use of RDTs, should thus be accompanied by health system strengthening strategies and activities.

## Competing interests

The authors declare that they have no competing interests.

## Authors' contributions

MIM- prepared the first draft, XB and JJ reviewed and edited the first and subsequent draft manuscripts. All authors read and approved the final manuscript.
